# *Akkermansia muciniphila* in infectious disease: A new target for this next-generation probiotic?

**DOI:** 10.1177/00368504241231159

**Published:** 2024-03-15

**Authors:** Jonathan M. Keane, Monica Cazzaniga, Cormac G.M. Gahan

**Affiliations:** 1 School of Microbiology, 8795 University College Cork, Cork, Ireland; 2 APC Microbiome Ireland, 8795 University College Cork, Cork, Ireland; 3 School of Pharmacy, 8795 University College Cork, Cork, Ireland

**Keywords:** *Akkermansia muciniphila*, infection, sepsis, inflammation, *Listeria*

## Abstract

The common gastrointestinal commensal *Akkermansia muciniphila* is a mucin-degrading bacterium that is greatly reduced in individuals consuming a high-fat diet. Increasing evidence from a variety of clinical and pre-clinical studies suggests that oral supplementation with *Akkermansia* can improve metabolic health and moderate systemic inflammation. We and others have demonstrated a role for *Akkermansia* administration in protection against infectious disease and the outcome from sepsis. Very recent studies have indicated the molecular mechanisms by which *A. muciniphila* may interact with the host to influence systemic immune-regulation and control of microbial pathogenesis. Here we consider recent studies which demonstrate the efficacy of this potential next-generation probiotic in animal models of *Salmonella* Typhimurium, *Listeria monocytogenes* and *Clostridioides difficile* as well as influenza virus and phlebovirus. The potential mechanisms by which *A. muciniphila* may influence local and systemic immune responses are discussed.

The increasing consumption of Western-style diets featuring an excess of calories from saturated animal fats, along with an increase in refined sugars and salt, is significantly impacting human wellbeing and longevity globally through influences upon adiposity as well as cardiovascular and immune health. Some components of this diet have the capacity to directly influence cellular metabolism, such as the long-chain fatty acids palmitic and stearic acid, which engage the NLRP3 inflammasome to drive low-grade inflammation in the host.^1^ Diet also has a significant influence on the gut microbiome, the composition of which impacts local inflammatory processes and gut barrier function through bacterial effectors such as short-chain fatty acids and bile acid modifications.^1^ Perhaps unsurprisingly, in animal models, Western diets induce susceptibility to infection with foodborne pathogens including *Listeria monocytogenes* and *Salmonella* Typhimurium.^2,3^ In the *Salmonella* model, a high-fat (HF) diet increases bile salt production, providing the pathogen (which is bile tolerant) with a competitive advantage over the microbiota.^3^ Our laboratory has demonstrated a role for diet in *L. monocytogenes* infection in mice, with an HF diet reducing resistance to both local and systemic infection alongside concurrent changes to microbiome composition and immune function.^2^ In a subsequent study, we show that prior oral administration of *Akkermansia muciniphila* provides protection against infection with *Listeria* in the HF dietary model system with an apparent reversal of specific effects of an HF diet.^4^ This commentary will contextualise those findings and point towards future work that is needed in this area if we are to understand and exploit the ability of the microbiota to protect against foodborne infection.

The role of the gut microbiota in resistance to *Listeria* infection has been demonstrated using germ-free animal models which generally demonstrate increased susceptibility to infection in comparison to conventionally raised animals.^5^ Mono-colonisation of gnotobiotic mice with *Lactobacillus* spp. prior to *L. monocytogenes* infection improved infection resistance, altered the expression pattern of interferon-stimulated genes in the host, and influenced the transcriptional response of the pathogen, suggesting potential mechanisms underpinning these findings.^6^ Antibiotic treatment of normal mice to reduce colonisation resistance prior to infection by *L. monocytogenes* significantly increases susceptibility to oral infection by the pathogen. Becattini et al.^7^ utilised this model to identify individual commensal taxa that are important in resistance to infection, and which could potentially act as next-generation probiotics in the prevention of disease.

Similarly, diet may be used as a modifier of the microbiome to break colonisation resistance and allow the discovery of commensal strains that actively impede foodborne pathogens.^3^ It is well established that an HF Westernised diet causes a reduction in abundance of *A. muciniphila*.^1^ In our initial studies we showed that abundance of *Akkermansia* spp. was impacted even following short-term dietary changes to an HF diet and that this correlated with increased susceptibility to both oral and systemic *L. monocytogenes* infection.^2^ We then set up an experimental model in which mice were given HF diets either with or without the addition of supplemental live *A. muciniphila* and showed that *Akkermansia* significantly improved resistance to subsequent oral and systemic *L. monocytogenes* infection.^4^ We demonstrated that provision of *Akkermansia* did not significantly impact the composition of the microbiome. Similarly, even though *Akkermansia* is reported to facilitate the production of the short-chain fatty acid butyrate, which is associated with positive health effects in the gut, we did not detect differences in the microbial metabolic products in the caecum or faeces between groups.^4^ This suggests that the protective effects of *Akkermansia* are not the result of large-scale shifts in the microbiota but rather the direct action of *Akkermansia* itself on the local environment of the gut.

Locally, in our model, *Akkermansia* reduced goblet cell numbers in the colon of mice fed with an HF diet,^4^ although this finding contrasts with another study which employed a longer period of HF dietary feeding.^8^ Goblet cells are a preferential site for cellular invasion by *Listeria* and this provides a potential local mechanism by which *Akkermansia* may enhance gastrointestinal, but not systemic, resistance to the pathogen.^9^ Additionally, *Akkermansia* may influence systemic infection resistance through a role in immune regulation, barrier function and homoeostasis. Indeed, a recent study showed that improved barrier function as a consequence of *Akkermansia* administration can protect against *Clostridioides difficile* infection in the colon.^10^ In our study, *Akkermansia* modified immune gene expression in the ileum (local) and liver (systemic), including regulation of genes encoding tumor necrosis factor-alpha (TNFα) both before and during *Listeria* infection.^4^ TNFα is well established as a key cytokine which enhances protection against *L. monocytogenes*. While microbiome-induced immune homoeostasis is primarily associated with immune suppression, up-regulation of TNFα expression by probiotic administration has been shown to influence hepatic, as well as gastrointestinal, innate immune signalling.^11^

The impact of gut *Akkermansia* interactions upon the systemic immune system is the subject of intense study. *Akkermansia* outer membrane protein *Amuc_1100* was shown to interact with enterocyte host Toll-like receptor (TLR) 2 and improve gut barrier function in mice.^8^ TLR2 is itself part of the TNFα signalling pathway in response to bacterial infection^12^ and *Akkermansia* Amuc_1100 has been shown to activate nuclear factor kappa B *in vitro*.^13^ Improved barrier function following *Akkermansia* administration is linked to a reduction in systemic lipopolysaccharide (LPS) with broad consequences for systemic immune regulation and macrophage infiltration into adipocytes in the context of obesity.^8^ Recent studies have demonstrated that administration of *Akkermansia* or Amuc_1100 locally in the oral cavity improved bone loss in an experimental model of inflammatory *Porphyromonas gingivalis*-induced periodontitis and had systemic effects upon inflammation.^14^ Furthermore, a tripeptide derived from *Akkermansia* (Arg-Lys-His; RKH) can prevent lethal sepsis in a variety of model systems.^15^ *Akkermansia* has also been shown to specifically modulate the adaptive immune response influencing T cell and antibody responses in the host; factors that potentially influence cell-mediated responses to pathogens and pathobionts.^16^ Indeed, the systemic immune-modulatory effects of orally administered live or pasteurised *A. muciniphila* have been linked to reduced pulmonary viral load in a mouse model of influenza infection.^17^ Another detailed study showed that *A. muciniphila* produces the β-carboline alkaloid harmaline which protects against phlebovirus (a member of the Bunyaviridae) through systemic immune modulation via a mechanism that involves enhancement of liver bile acid production and subsequent immune-signalling via transmembrane G-protein coupled receptor-5 (Figure 1).^18^

**Figure 1.**
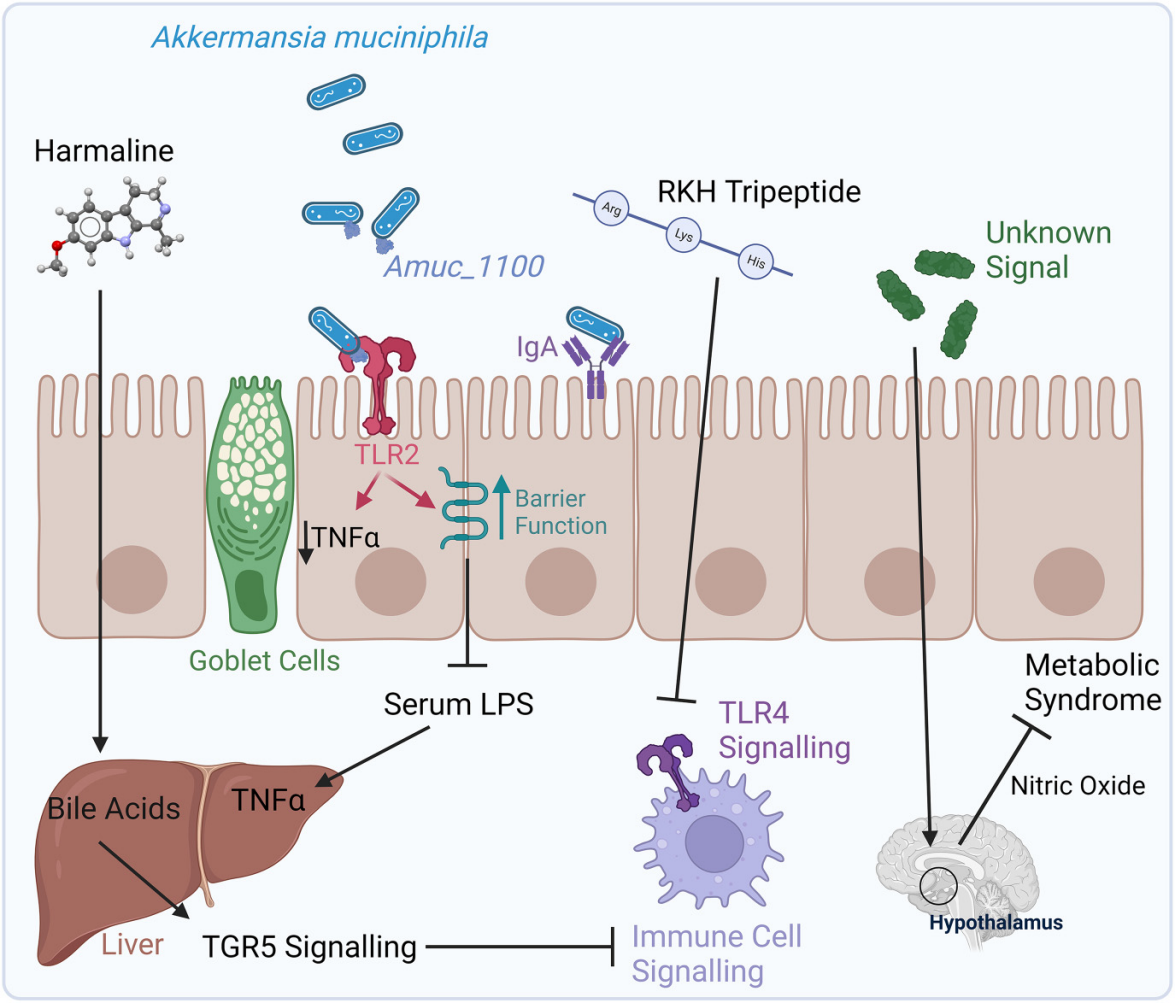
*Akkermansia muciniphila* mediates its effects on a local and systemic level. In the intestine, *Akkermansia* influences the number of goblet cells and down-regulated TNFα expression.^4^ Its outer membrane protein, Amuc_1100, was shown to activate TLR2 and improve barrier function with an associated reduction of systemic LPS (a key driver of systemic inflammation),^8^ while it may also modulate immune homoeostasis by influencing IgA and local adaptive immune responses.^16^ *A. muciniphila* has the capacity to influence systemic immune parameters via a variety of proposed mechanisms. *A. muciniphila* produces the β-carboline alkaloid harmaline which protects against phlebovirus infection in the lungs through a proposed mechanism that involves up-regulation of bile acid synthesis in the liver and modulation of immune signalling via the TGR5 bile acid receptor.^18^ The tripeptide RKH is produced by *Akkermansia* and has been shown to reduce TLR4 signalling pathways resulting in moderation of potentially lethal sepsis.^15^ Finally, *A. muciniphila* is known to activate the enteric nervous system and impact signalling to the hypothalamus,^21^ although the implications of this interaction for immune homoeostasis are currently unclear. Figure was generated using Biorender. TGR: transmembrane G-protein coupled receptor; TLR: Toll-like receptor; TNFα: tumor necrosis factor-alpha.

*Akkermansia* also displayed systemic effects in pathologies other than infection. It ameliorated harmful phenotypes associated with obesity by modulating host urinary metabolomic profiles and intestinal energy absorption.^8^ *Akkermansia* also appears to mitigate the negative effects of systemic interferon gamma on glucose metabolism, with significance for diabetes.^19^ *Akkermansia* has even been shown to ameliorate chronic stress-induced depressive symptoms in mice, implicating it in the gut–brain axis,^20^ and recent evidence shows that gut motility and glycaemic control is influenced through *Akkermansia* triggering gut neurons to influence hypothalamic nitric oxide signalling.^21^ All of this evidence demonstrates that the physiological influence of *Akkermansia* goes beyond the local environment of the gut and can moderate systemic immune-metabolic-inflammatory pathways with implications for host health. Some of these potential mechanisms are outlined in Figure 1.

Whilst we examined *Akkermansia* administration and *Listeria* infection in the context of an HF diet, we have not yet determined the potential impact in mice fed normal chow or a low-fat diet. There remains the possibility that the effects are diet-dependent and result from amelioration of the detrimental effects of HF diet by *Akkermansia* administration in our model. Furthermore, potentially harmful effects of *Akkermansia* overabundance have been noted and discussed previously.^22^ Mucous degradation by *Akkermansia* in the gut stimulates enhanced mucous secretion benefiting host barrier function and impacting glucose metabolism and adiposity (reviewed in Chiantera et al.^22^). However, mucous degradation may potentially damage the gut barrier, and *Akkermansia* administration has been associated with enhanced inflammation in mouse models of inflammatory bowel disease and enhanced tumorigenesis in a model of colon cancer.^22^ In a reduced complexity microbiota model (the SHIUMI model), the addition of *Akkermansia* actually promoted *Salmonella* infection and exacerbated inflammatory scores.^23^ However, the effects may be dependent on microbiota complexity as administration of both live *Akkermansia* or a pasteurised preparation reduced burden of *Salmonella* infection in a conventionally raised murine infection model.^24^

In summary, studies have demonstrated a capacity for *Akkermansia* to reduce infectious load in *L. monocytogenes, C. difficile*, *Salmonella* and virus-infection models through influences upon gut barrier function and the local and systemic immune system.^4,10,17,18,24^ However, further studies are required to investigate mechanisms in more detail, to further examine the systemic immune-modulatory effects, and to determine impacts against other infectious agents. It is notable that the safety and clinical efficacy of live and pasteurised *A. muciniphila* in reducing several parameters of metabolic syndrome in humans was demonstrated as part of clinical trial NCT02637115.^22^ Furthermore, pasteurised *A. muciniphila* has been classified as a novel food by the European Food Safety Authority, permitting its sale and consumption by the public outside of a clinical setting.^22^ This establishes *A. muciniphila* as a next-generation probiotic, supplementing the established genera of *Lactobacillus* and *Bifidobacterium*. Our work demonstrates potential impacts of *Akkermansia* upon infection and adds to the growing armoury of commensal strains that may have the potential to limit infectious disease. Together with the work of others, this suggests a novel approach to the prevention and limitation of infectious disease through understanding and implementing alterations to the microbiota.^3,25,26^
